# Personnel time requirements for mobile chest X-ray screening for TB

**DOI:** 10.5588/ijtldopen.25.0254

**Published:** 2025-10-10

**Authors:** T.S. Johnson, J. Kakeeto, D. Isooba, S. Birabwa, J. Magezi, W. Kamya, R. Okura, I. Naluyima, A. Nalutaaya, P.J. Kitonsa, E.A. Kendall, A. Katamba, D.W. Dowdy

**Affiliations:** ^1^ Johns Hopkins Bloomberg School of Public Health, Epidemiology, Baltimore, MD, USA;; ^2^ Walimu, Uganda Tuberculosis Implementation Research Consortium, Kampala, Uganda;; ^3^ Uganda Tuberculosis Implementation Research Consortium, Makerere University, Kampala, Uganda;; ^4^ Department of Medicine, Johns Hopkins University School of Medicine, Baltimore, MD, USA;; ^5^ Department of Internal Medicine, Clinical Epidemiology and Biostatistics Unit, Makerere University College of Health Sciences, Kampala, Uganda.

**Keywords:** tuberculosis, human resources, diagnosis, artificial intelligence, active case finding

## Abstract

**BACKGROUND:**

Community-based active case finding (ACF) using chest X-ray (CXR) is effective for early TB detection, but implementation is limited by high resource demands.

**METHODS:**

We assessed human resource needs for two ACF strategies – community-based and facility-adjacent – using mobile CXR with computer-aided detection during a cluster randomised crossover trial in peri-urban Uganda. Tuberculin skin testing (TST) was offered, with referral to preventive therapy for those with positive TST but negative TB evaluation. We conducted time-and-motion observations of three- and four-member screening teams over 90 days (July 2023–April 2024). We estimated staff time per key screening outcome, including time per positive Xpert result and per positive TST reading.

**RESULTS:**

Given an average yield of 6.9 cases per 1,000 individuals screened, three-member (four-member) screening teams collectively spent 65.0 (80.2) person-hours per positive Xpert result, or 26.7 (32.9) person-hours per positive TST read. Staff performed a diverse range of activities, of which the most time-consuming were initial intake (community-based: 2.0 h/day, 23% of time; facility-adjacent: 2.7 h/day, 31% of time) and participant counselling (1.6 h/day, 18%; 2.0 h/day, 22%).

**CONCLUSION:**

TB ACF requires substantial human resources for implementation. National TB Programs should carefully consider personnel requirements when planning and scaling these programmes.

TB remains a significant contributor to global morbidity and mortality, despite the existence of several effective tools to screen for and treat TB infection and disease.^1^ The End TB Strategy calls for an investment in research to optimise implementation and impact of existing evidence-based interventions, including community-based active case finding (ACF).^2,3^ Although ACF scale-up is a priority at the national level in many countries, there is a lack of comprehensive data describing the human resource requirements for health care workers supporting TB control efforts in low- and middle-income countries.^4–6^

Previous studies have demonstrated the effectiveness of ACF using chest X-ray (CXR) as a TB screening tool.^7–10^ TB programmes have historically opted to implement verbal symptom screening over community-based CXR due to the intensive human resource and infrastructure requirements associated with CXR.^11,12^ Computer-aided detection (CAD) uses artificial intelligence (AI)-powered software to analyse chest radiographs for TB-related abnormalities, providing probability scores for screening or triage and offering an efficient alternative to CXR interventions that require clinicians to interpret each image.^13^ Other studies have established the accuracy of CAD for screening, but there is a need for implementation data to guide integration into workflows of TB programmes in high-burden settings.^12,14^

The aim of this study was therefore to describe and analyse the human resource requirements for implementation of TB ACF using CXR and CAD in two different types of screening location in peri-urban Uganda. Specifically, our objectives were to 1) define the time spent in each activity required to implement the screening intervention and 2) determine the staff time necessary to produce specific screening outcomes (e.g., time per positive Xpert result).

## METHODS

From 1 July 2023 to 12 April 2024, we conducted a time-and-motion (TAM) observational study as part of an ongoing cluster randomised crossover trial of ACF for TB. The trial, conducted in eight health-facility catchment areas within 200 km of Kampala, Uganda, is investigating the effectiveness of two strategies for implementing mobile CXR screening for TB (Clinicaltrials.gov: NCT05285202). The ‘community-based’ strategy sends mobile CXR units to various public locations in portions of the catchment area that are believed to have the highest prevalence of undiagnosed TB; locations for screening are selected in consultation with staff at local health facilities. The ‘facility-adjacent’ strategy conducts screening in a fixed location adjacent to the health facility that defines the catchment area. Catchment-area clusters are randomised to alternating strategies for 4 months at a time (interspersed with 4-month ‘washout’ periods); the primary trial outcome is the number of people starting treatment for TB at the catchment-area level. Consenting participants ≥15 years of age are offered CXR, with analysis by a CAD programme (qXR, Qure.ai) that typically generates a result within 2 min. Participants whose qXR scores are above a pre-specified threshold (0.1 during this TAM substudy) are offered Xpert® Ultra testing (Cepheid, Sunnyvale, CA, USA). Asymptomatic, HIV-negative participants are also offered tuberculin skin testing (TST) on the day of screening.

### TAM analysis

TAM studies provide detailed insights into how tasks are performed, helping to identify inefficiencies in service delivery.^15^ TAM involves direct observation of health care workers, measurement of time spent on each task, and analysis of workflows.^16^ Findings from TAM studies have been applied in numerous ways to inform programme implementation, including optimising community health worker (CHW) activities in Tanzania, improving hygiene practices among birth attendants in Zanzibar, and recommending task-shifting to enhance efficiency in Uganda’s HIV clinics.^17–19^ In our study, we applied this approach by directly observing staff activities as they implemented each screening intervention. Specifically, each team consists of three to four staff members. One team member (‘counsellor’) counsels participants before referring participants to a second (‘X-ray operator’) who performs the CXR and refers back to the counsellor. The counsellor discusses CXR results with participants and, depending on the qXR score and symptom status, coaches participants on sputum collection and/or offers TST. The counsellor also assists participants when they return for results. The additional team members (CHW and driver) primarily support participant recruitment and logistical tasks, with the driver working only at community-based sites. Counsellors and X-ray operators are generally cross-trained; as such, staff occasionally perform additional duties beyond their primary responsibilities if appropriately trained.

### Measures and data collection

We employed a rotating observation schedule in which we assigned the teams at each of 11 sites (6 community-based and 5 facility-adjacent) to specific observation days, such that teams at each type of site were observed for 45 days over the course of the evaluation (90 days total, Table) with balance across site types in day-of-week and season-of-observation. Prior to each observation day, a single research assistant (RA) randomly selected one staff role to observe from among all staff on the team expected to work the following day. Throughout the day of observation, the RA used a tablet-based tool to record start and stop times for each discrete activity performed by the selected staff member, applying a set of activity codes developed in consultation with the Ugandan research team and pilot-tested over 2 weeks. We defined time ‘actively working’ as the sum of all daily activity times, excluding unstructured time. We also tracked the number and proportion of eligible participants completing each step of the screening cascade to estimate the average number of screening activities carried out daily at each site type and the probability of completing each step of the screening cascade (e.g., proportion of screened participants submitting sputum and proportion of sputum specimens Xpert-positive) by site type. All study data were collected and managed using REDCap electronic data capture tools hosted at Johns Hopkins University.^20,21^

**Table.** Staff time required per day for facility-adjacent and community-based screening for TB using chest X-ray.

Staff role^**A**^	Site type	Days observed	Total observed minutes	Mean active minutes per day, HH:MM (Std Dev)^**B**^
Counsellor	Facility	15	4,889	6:38 (2:26)
	Community	16	5,028	7:31 (1:39)
X-ray operator	Facility	24	6,558	5:06 (1:47)
	Community	14	4,716	5:45 (1:33)
CHW	Facility	7	2,035	4:45 (0:54)
	Community	6	1,235	4:12 (1:22)
Driver	Community	9	1,539	3:57 (1:11)

A Facility-adjacent teams typically employed three people per site: one counsellor, one X-ray operator, and one community health worker (CHW), whereas community-based teams typically also employed a driver. The CHW often did not work the full day at the screening site, and while the driver in community-based teams often assisted with daily preparation and tear-down, this person was not generally working for substantial periods of the day.

B Teams worked an average of 5 h and 22 min (SD = 1:38) in community-based sites and 5 h and 29 min (SD = 1:00) in facility-adjacent sites. Counsellor time exceeds these estimates in both cases because of periods when the X-ray operator had finished work before the counsellor or was waiting for additional participants who were being screened by the counsellor.

### Analyses

The primary outcome of this analysis was the total staff time (in person-hours) required to achieve each of several pre-specified screening outcomes. Specifically, we estimated the staff time investment required per CXR performed and read, per positive sputum result, and per positive tuberculin skin test (TST) read, by constructing samples of 30 simulated workdays and calculating the mean time worked per outcome achieved across each sample. To estimate the variance in these results, we utilised a bootstrapped simulation approach, assuming a three-member team (counsellor, X-ray operator, and CHW, reflecting the usual staffing at our facility-adjacent sites), and separately assuming a four-member team (including a cross-trained driver). Given that results of the primary trial remain masked, we assumed that outcomes were similar across site types and team sizes in this analysis. We constructed the sets of 30 simulated workdays by sampling with replacement one day at a time from each staff role’s sample of observation-days. To estimate the corresponding number of people screened, we likewise sampled without replacement the number of people screened each day across 30 random screening days from the full sample of participant screening data. To estimate the prevalence of positive MTB or TST results among the total number screened in that 30-day sample, we applied a sequence of binomial probabilities derived from the full sample of empirical screening data, resulting in a simulated number of positive results for the population screened over that hypothetical 30-day span. We performed 1,000 iterations of sampling, with each iteration corresponding to the total staff time worked across 30 observation-days (including three or four staff) and screening outcomes derived from 30 paired screening days. We defined 90% uncertainty ranges as the 5th and 95th percentiles across all 1,000 (30-day) simulations.

Secondary outcomes included assessments of staff time allocation across a hypothetical screening day and the distribution of staff time throughout the hours of a typical workday, comparing each outcome between screening strategies (i.e., community-based vs. facility-adjacent). For these outcomes, we first calculated the mean daily time each staff role spent actively working. We allocated individual activity observations into three categories – participant interaction (clinical or administrative) and support activities – and calculated the mean daily time spent on each category across staff roles. To estimate the proportion of total staff time (i.e., across all staff present at the site) dedicated to each activity category during each hour of a typical workday, we generated a ‘typical’ workday, separately for community-based and facility-adjacent sites, by calculating the mean total time spent on each activity category within each hour of the day. Within each hour of the workday, we scaled the time spent on each activity relative to the number of observation-minutes made during each hour across the full sample of observations.

We performed all statistical analyses using R Statistical Software (Version 4.3.2; R Core Team 2023).

### Ethical statement

The Ethics Review Committees of the Makerere University School of Public Health (study reference number: SPH-2021-181) and Johns Hopkins School of Medicine (IRB protocol number: PE00012778) approved the study.

## RESULTS

On average per day, staff members spent 4 h and 50 min (SD = 1.94 h) actively working at community-based sites, versus 5 h and 33 min (SD = 2.06 h) at facility-adjacent sites. Table provides the breakdown of time spent according to staff role.

**Figure 1.**
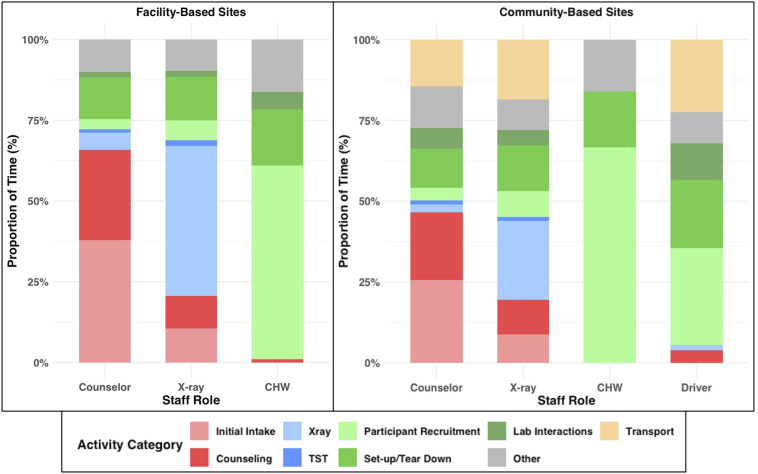
Percentage of daily staff time spent on activities at facility-adjacent and community-based mobile chest X-ray screening sites. Staff spent time on non-clinical activities with participants (red), clinical activities with participants (blue), and support activities (green). Activities coded as ‘other’ included blood pressure measurements, providing additional assistance to participants with sputum expectoration, and programmatic and research-specific administrative work. Mean times for each activity type were calculated using the total observation time for each staff role, and the percentages were calculated using the mean total daily active work time for that staff role (Table). Wait time, time spent idle due to site disruptions, and break time were also observed but are omitted from the figure. Community-based sites were staffed with four staff members (counsellor, X-ray operator, community health worker [CHW], and a driver), while facility-based sites were staffed with three staff members (counsellor, X-ray operator, and CHW).

**Figure 2.**
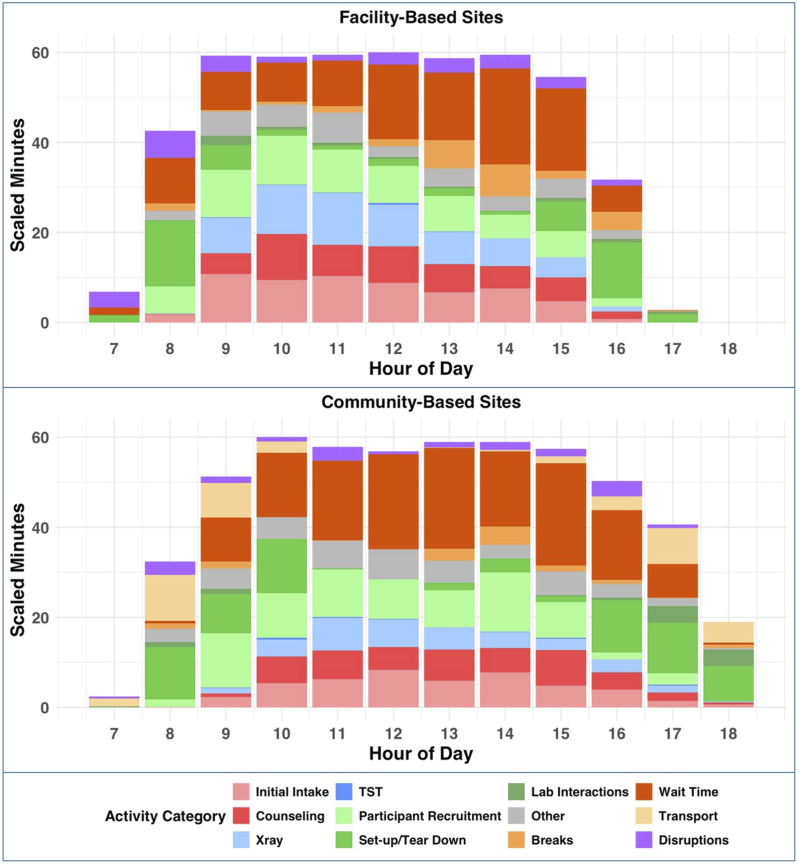
Distribution of activities carried out by mobile chest X-ray screening staff throughout the day at community- and facility-based sites. The plots show the average minutes spent on each activity category on an hour-by-hour basis, divided by the number of staff working that hour. Activities are categorised as participant interaction – administrative interaction activities are displayed in shades of red and clinical participant interaction activities are displayed in shades of blue. Support activities are displayed in shades of green. See the Appendix for a complete listing of activities. The top plot shows the normalised distribution of time spent on activities at facility-based screening sites, and the bottom plot shows the same analysis at community-based screening sites.

Counsellor time was largely spent on initial intakes (31% and 23% of counsellor time at facility-adjacent and community-based sites, respectively) and follow-up counselling (22% and 18%, respectively) (Figure 1). X-ray operators mostly focused on direct X-ray performance, requiring 32% of X-ray operator time at facility-adjacent sites and 21% at community-based sites. CHWs spent 61% of their active work time (2.95 h/day, SD = 0.89) in facility-adjacent sites and 66% (2.76 h/day, SD = 1.19) in community-based sites on participant recruitment. Site set-up and tear-down required between 10% and 12% of individual staff members’ daily work time. Teams of staff supporting community-based sites spent an average of 1.13 h (SD = 0.05) in transport. Participant interaction (i.e., intake, counselling, and X-rays) peaked at 10 am at facility-adjacent sites, while remaining roughly constant from 10 am to 4 pm at community-based sites (Figure 2).

**Figure 3.**
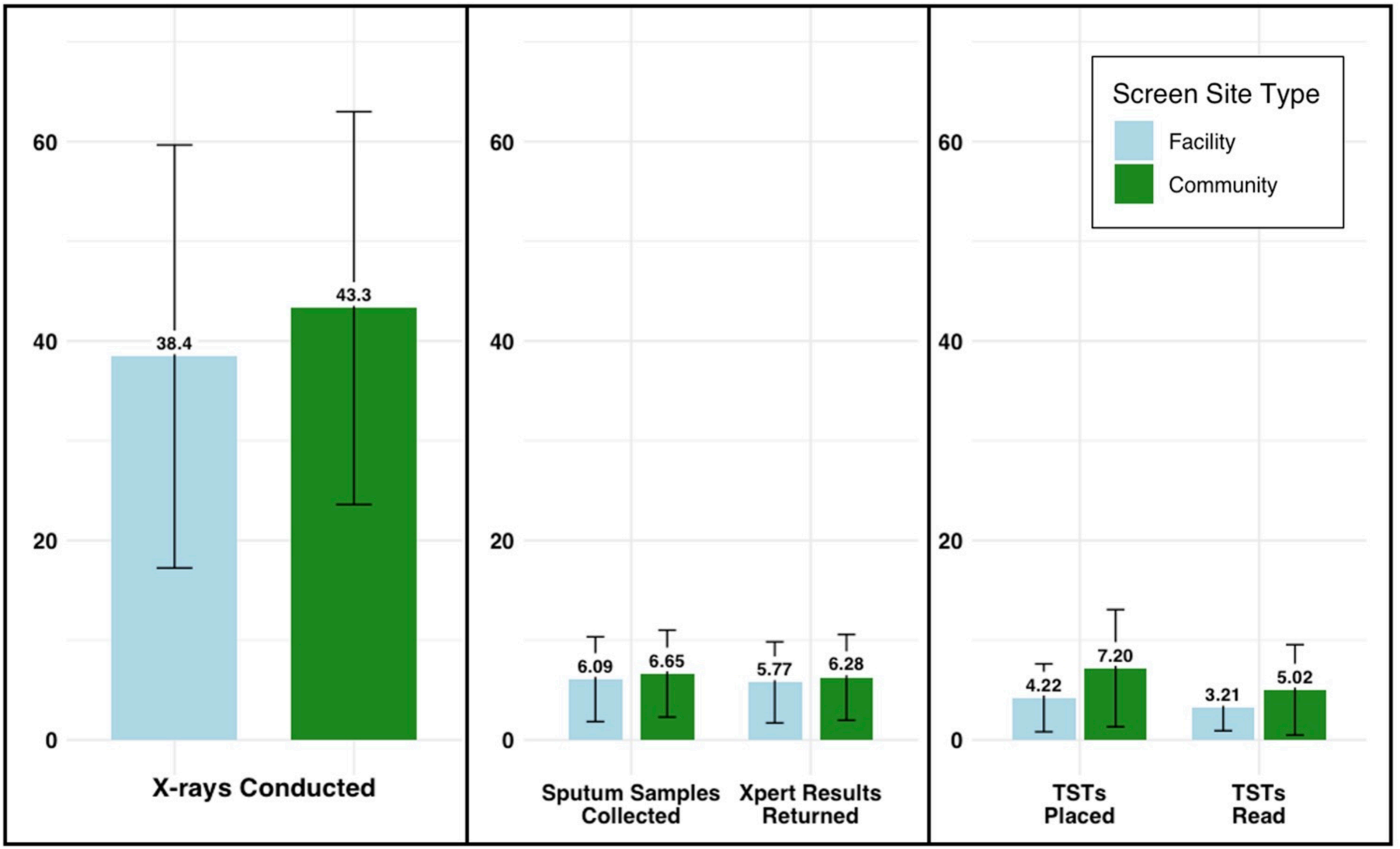
Mean number of activities conducted per day at facility-adjacent and community-based mobile chest X-ray screening sites. In this intervention, all participants underwent an initial X-ray, with the daily screening volume shown in the first panel. The middle panel illustrates the daily volume of participants who completed the steps of providing a sputum sample and receiving their results after their X-ray indicated presumptive TB. The third panel provides corresponding estimates for all participants whose X-ray results did not indicate presumptive TB, who proceeded to tuberculin skin test (TST) placement and returned for TST reading after 48–96 h. Blue bars illustrate numbers in facility-adjacent screening, whereas green bars denote community-based screening; error bars indicate standard deviations.

Over 1,467 site-days of screening, 61,014 participants received a CXR screen (mean 41.6 per site per day). Sputum was collected from 9,585 participants (6.53 per site-day), resulting in 440 positive Xpert Ultra results (0.30 per site-day) This resulted in a prevalence of 6.9 positive cases per 1,000 individuals screened. Over 677 screening days in which TST was offered, TSTs were placed for 3,930 participants (5.81 per site-day), 2,181 participants returned for TST reading (3.22 per site-day), and 516 participants had a positive TST result read (0.76 per site-day). The daily screening volume was largely similar across both types of site, with the exception that TST placement and reading were more frequently completed at community-based sites (Figure 3).

**Figure 4.**
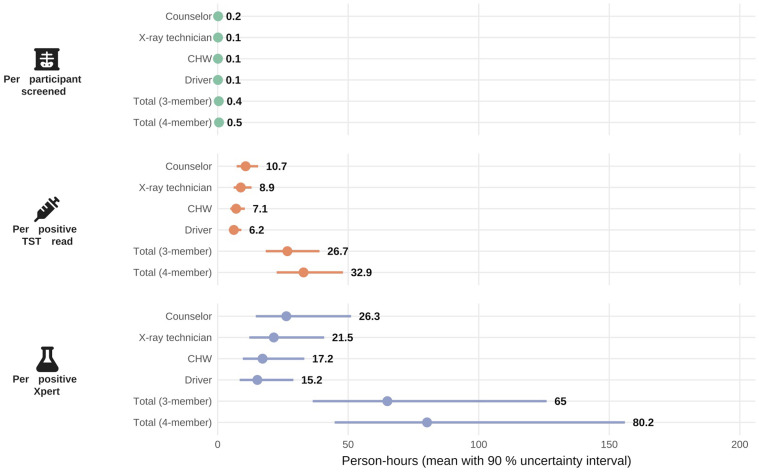
Total staff time required (in person-hours) per screening result by staff role and across the full screening team. Mean person-hours (dots) with 90% uncertainty intervals (bars) are shown for each staff role – counsellor, X-ray technician, community health worker (CHW), and driver – and for the full three- and four-member screening teams. Staff time estimates were generated by bootstrapping: active work minutes for each role were summed across 1,000 simulated datasets (30 workdays each), and the interval limits are the 5th and 95th percentiles of those simulations. Results are presented separately for participants screened, positive tuberculin skin test (TST) readings, and positive Xpert MTB/RIF results. Staff time for the TST outcome is calculated only on days when at least one TST was read, because prolonged tuberculin stock-outs intermittently halted TST placement and reading.

Each participant screened required 24.9 person-minutes or 0.42 (90% uncertainty range: 0.37–0.46) person-hours of total active work on average across all role types (Figure 4). For a three-person screening team, intervention activities generated one positive Xpert result for every 65.0 h (36.4–126.0) of staff time and, simultaneously, one positive TST result for every 26.7 h (18.4–39.0) of staff time.

## DISCUSSION

We examined the time required to implement two strategies for implementing a mobile CXR screening intervention for TB in peri-urban Uganda. We estimated that more than 60 person-hours (i.e., more than a week and a half of full-time-equivalent [FTE] work) were required to detect one participant with TB disease. We estimated that screening one person for TB would take an average of 25 person-minutes, with over one third of that time dedicated to support activities such as daily site set-up and tear-down, participant recruitment, and interfacing with laboratory staff. These findings highlight the substantial human resource investment required for TB active case finding. Based on these estimates of human resource requirements, for a TB programme to detect 100 people with TB through similar population-based screening, it would need to budget more than three FTEs for a full year. These findings are consistent with a review of determinants of ACF success in other low- and middle-income settings, which also identified human resource requirements as a major barrier to implementation.^22^ A survey of National Tuberculosis Program managers also indicated that the overburdening of health care workers is a perceived risk associated with ACF investment.^6^

Our findings could inform future implementation of similar interventions in a programmatic setting. The volume of screening participants was steady throughout the day at community-based sites, whereas the volume at facility-adjacent sites peaked in the morning and then decreased throughout the day. Programme planners could utilise these data to develop activity schedules and allocate staff time for each strategy – for example, identifying other non-TB-related activities that could be performed by staff in facility-adjacent sites in the afternoons. While counsellors and X-ray operators efficiently managed participant volume, CHWs and drivers experienced more down time and could potentially support participant-interaction tasks that do not require specialised training.

This study has important strengths. TAM studies that employ continuous and independent observation of health worker activities are considered the most reliable method of studying clinical workflows.^23^ By integrating this study prospectively within an ongoing cluster randomised implementation trial, we were able to collect dependable data across an extended period of observation days that are likely representative of time use across a full year. Despite these strengths, however, there are also key limitations associated with this analysis. Some attributes of the research setting may limit generalisability to a programmatic context. If TB screening was integrated into routine clinical practice, staff would need to balance the screening intervention with other responsibilities (e.g., clinical management of conditions other than TB) – but might also achieve additional efficiencies in doing so. We also were not fully able to separate activities that would be required programmatically from those that were purely for research purposes and may have overestimated the time required for some activities. Conversely, staff implementing the intervention in a controlled research setting such as this may be more efficient than routine clinical staff, possibly due to higher motivation from better salaries, more comprehensive training, supportive supervision, and fewer competing priorities. Future studies could apply similar methods to evaluate ACF in programmatic settings. Our estimates of the time required per screening outcome also reflect underlying factors such as the prevalence of TB, the availability of sufficient materials and supplies to collect sputum and administer TST, and willingness of participants to return to the screening site for results. As such, our estimates should be generalised to other settings only with caution. Finally, because of the combined nature of the intervention, estimates of time investment per positive MTB include time that staff spent on TST placement and readings, so the estimates may be slightly inflated. However, our results show that time spent on TST was minimal, so this effect is likely negligible.

## CONCLUSION

We demonstrate the time required to deliver population-based mobile CXR screening for TB in a low-income-country setting with a TB prevalence of 400 per 100,000 people, estimating that one-and-a-half to two full person-weeks of effort are needed per positive Xpert Ultra result obtained. Our findings highlight the importance of careful planning and resource allocation in ensuring the efficiency of TB screening. The substantial time investment required to achieve key screening results underscores the need for integrated approaches that maximise the use of available resources. Programme planners should utilise time and motion data to inform strategic planning for resource-intensive TB interventions, particularly to strengthen the case for investment in active TB case finding in high-burden settings.
